# Plant-Based versus Animal-Based Low Protein Diets in the Management of Chronic Kidney Disease

**DOI:** 10.3390/nu13113721

**Published:** 2021-10-22

**Authors:** Carmen-Antonia Mocanu, Tudor Petrisor Simionescu, Andreea Elena Mocanu, Liliana Garneata

**Affiliations:** 1 Department of Internal Medicine and Nephrology, “Carol Davila” University of Medicine and Pharmacy, 050474 Bucharest, Romania; lilianagarna@yahoo.com; 2 Department of Nephrology, “Dr. Carol Davila” Teaching Hospital of Nephrology, 4 Calea Grivitei, Sector 1, 010731 Bucharest, Romania; tudorsimi@yahoo.com (T.P.S.); andreeamocanu91@gmail.com (A.E.M.)

**Keywords:** chronic kidney disease, low-protein diets, vegetarian diets, plant-based diets, management of CKD, dietary intervention in CKD

## Abstract

Recent data reiterate low-protein diets (LPDs) as cornerstones in the conservative management of chronic kidney disease (CKD). The reduction in proteinuria, better blood pressure control and the reduction in the rate of decline in kidney function with LPDs were reported, both in non-diabetics and diabetics patients. Supplemented, vegetarian, very-low-protein diets (sVLPD, 0.3 g/kg-day) could postpone kidney replacement therapy (KRT) initiation, mainly through the better control of metabolic disorders of advanced CKD in non-diabetic patients. Plant-based diets could ameliorate gut microbiota and appear to be superior to mixed hypoproteic diets in treating advanced CKD: better control of nitrogen balance, acid-base metabolism and bone mineral disorders. Vegetarian diets generate fewer uremic toxins and reduce salt intake and acid overload. At the same time, they can improve lipid metabolism, providing a high ratio of unsaturated to saturated fatty acids, as well as insulin resistance.

## 1. Introduction

Chronic kidney disease (CKD) is a worldwide healthcare problem, both in the predialysis stages and during kidney replacement therapy (KRT). With a prevalence of 10% in the general population [1], it significantly impacts patients’ morbidity, mortality and quality of life, as well as healthcare costs.

Therefore, it is important to focus on the possibility of reducing the progression of CKD and postponing RRT through a multifactorial approach, including dietary protein restriction.

The largest trial which examined the efficacy of nutritional intervention in CKD, the Modification of Diet in Renal Disease study group (the MDRD study), yielded controversial results [2]. Upon a secondary analysis, a mild improvement in the rate of decline in kidney function was noticed with LPDs compared to a normal-protein diet. Concomitantly, concerns about possible malnutrition were raised [3]. The long-term follow-up of these results showed no difference in kidney survival, except in poorer patients’ survival in the supplemented very-low-protein diet (sVLPD) group [4]. However, several points from the MDRD study were debated: the use of the achieved versus prescribed protein intake in the analysis, as well as differences in the selection criteria and in the monitoring schedule [4].

In recent years, there is increasing evidence that low-protein diets (LPDs) can favorably influence the decline in kidney function [5,6,7].

## 2. Low-Protein Diets in CKD

**The recommended protein intake**. The KDOQI Clinical Practice Guidelines for Nutrition in CKD recommends to patients with metabolically stable CKD stage 3+, under close monitoring, protein restriction and in some circumstances supplementation with ketoanalogues of essential amino acids. The effect of this type of dietary intervention seems to reduce the risk of mortality and progression to the final stage of CKD, with an improvement in patients quality of life [8]. In this regard, for non-diabetic patients is recommended a protein intake of 0.55–0.6 g/kg-day (representing a LPD) or a protein intake of 0.28 to 0.43 g/kg-day supplemented with ketoanalogues (representinga sVLPD) [8]. Diabetic patients have a recommendation to maintain a protein intake of 0.6–0.8 g/kg-day with the additional optimization of blood glucose control [8].

Reducing protein intake may affect the nutritional status of patients at risk of protein-energy wasting. However, Western patients who consume processed red meat have a protein intake above the optimal level considered necessary to achieve a nutritional balance (1.35 g protein/kg-day versus 0.8 g/kg-day). Moreover, a further reduction in protein intake (0.3–0.4 g/kg-day) supplemented with ketoanalogues it can also provide the required daily dose of essential amino acids [8]. Ketoanalogues are organic compounds containing groups of carboxylic acid and groups of ketone which, by transamination, form amino acids. Therefore, ketoanalogues can be used preferentially to amino acids to supplement the diet in patients with CKD. Most ketoanalogues contain four ketoacids of essential amino acids (isoleucine, leucine, phenylalanine and valine), a hydroxy-acid of methionine, and four essential amino acids for patients with CKD (tryptophan, threonine, histidine and tyrosine) [9].

The major concern when it comes to the LPDs, especially in vegetarians, is the risk of malnutrition, since very restrictive diets with limited food sources can struggle to reach the recommended daily dose of certain amino acids, vitamins or even energy intake. 

There are also effects on the kidney function. The dietary protein intake in CKD has been investigated in several recent studies, suggesting that Western diets, with an increased content of processed red meat, were associated with the accelerated progression of CKD, three times higher than normally expected (−3 mL/min/1.73 m^2^) and increased proteinuria [10,11].

In the Atherosclerosis Risk in Communities (ARIC) study, in 14,882 patients with a median estimated glomerular filtration rate (eGFR) above 60 mL/min/1.73 m^2^ at baseline, consuming mainly processed red meat was associated with up to a 25% reduction in kidney function [12]. In contrast, patients in the Dietary Approaches to Stop Hypertension (DASH) diet, with a low meat consumption and increased vegetables, fruits and low-fat consumption had a 14% lower risk of developing CKD [13].

A high-protein intake causes the vasodilation of the afferent arteriola, increasing the intraglomerular pressure, and thus the progression of glomerulosclerosis [11]; while low-protein diets cause the vasoconstriction of the afferent arteriola, decreasing the intraglomerular pressure and the rate of CKD progression [14].

Clinical studies showed the additional benefits of LPDs in CKD: the amelioration of nitrogen balance, metabolic acidosis, calcium-phosphorus metabolism, insulin resistance and of blood pressure control [15,16,17].

**Kidney and patient survival.** Several studies showed LPDs to be beneficial both for patient and kidney survival in adherent patients. In 1985, Rosman et al. demonstrated that patients who were compliant to LPD had a better survival rate compared to the patients who did not follow a dietary intervention (55 versus 40%) [18]. In this regard, other studies also showed a favorable effect on kidney survival in patients adherent to LPDs (90 versus 73%) [19]. Another randomized clinical trial aimed to assess the patient and kidney survival in non-diabetic patients with a stable CKD stage 4+, in which the patients were randomized to a LPD (0.6 g/kg-day) and a sVLPD (0.3 g/kg-day + 1 tb/5 kg-day of ketoanalogues) [20]. The achieved protein intake was assessed using both food dairy and the urinary urea in the 24 h urine collection [21]. The patients continued to follow the initial intervention for a median surveillance of 10 years. There were significant differences between the groups both in patient survival (89% in sVLPD versus 60% in LPD) and in kidney survival (51% patients from sVLPD group did not need KRT versus 93% in the LPD group) [20]. However, since in this study there were two main differences between the groups, i.e., the level of achieved protein intake, as well as the vegetarian content of the diet, the role of the vegetarian diet per se was difficult to assess.

**LPDs in Diabetic Kidney Disease.** Diabetic kidney disease is one of the most common etiologies of CKD, characterized by significantly higher proteinuria, and thus a more rapid decline in kidney function when compared to other kidney diseases [22]. Persistent hyperglycemia, insulin resistance or insulin deficiency induce metabolic disorders, promoting oxidative stress and advanced glycosylation compounds. This causes endothelial dysfunction, podocyte dysfunction, as well as mesangial and tubular abnormalities [23]. An important mechanism in the production of diabetic nephropathy that deserves additional attention is the stimulation of the renin-angiotensin-aldosterone system [24]. LPDs seem to interfere with the angiotensin–aldosterone system, and thus decrease the intraglomerular pressure. The main positive effects are seen in reducing proteinuria [3,25,26], but also in postponing KRT [27].

The multifactorial approach is currently recommended in diabetic patients; therefore, there are limited data regarding the effects of a low-protein diet per se in a patient with diabetic kidney disease, but the few studies that address the role of the diet are in favor of low-protein diets, mostly vegetarian, primarily because of the hemodynamic effect of such a diet [17,26]. More than that, diabetic patients who were adherent to vegetarian LPD had a better control of glucose metabolism because of the decreased insulin resistance [28].

## 3. Plant-Based Diets in CKD

Mediterranean diets, rich in vegetables, fruits and cereals were associated with better survival in patients with CKD as compared to Western diets [29]. Patients who consume a high content of processed food seem to have an increased cardiovascular and all-cause mortality and a higher incidence of neoplastic diseases compared with patients who do not consume a high content of processed food [30,31].

Vegetarian and vegan diets are increasingly used in patients with chronic diseases, including CKD, especially to facilitate the integration of LPDs [32,33]. Vegan and vegetarian diets typically contain 0.6–0.8 g/kg-day of proteins and seem to attenuate hyperfiltration, through a lower bioavailability of plant proteins as compared with the animal ones [34,35,36].

In addition, vegetarian and vegan diets are rich in vitamins.

Diets based solely on plant proteins were considered for a long time nutritionally inadequate, mainly because of the potential deficit of essential amino acids found in animal proteins. In fact, all foods contain amino acids and vitamins in different proportions; an appropriately balanced vegetarian diet achieves the same amino acid and vitamins content as the animal-based proteins [37,38,39].

For the assessment of the nutritional value of food the Protein Digestibility Corrected Amino Acid Score (PDCAAS) can be used [40]. This score has been used to measure the quality of proteins from aliments or groups of aliments with standard amino acid profiles. The score compares the amount of the essential amino acids from a certain aliment to a scoring pattern based on the requirements of 2- to 5-year-old children, in whom there are standardized, maximum nutritional requirements [41]. The main limitation of this score is that it does not take into account the levels of some anti-nutrients, such as phytic acid or trypsin. Since 2013, the Food and Agriculture Organization of the United Nations proposed another system for measuring the quality of proteins, the Digestible Indispensable Amino Acid Score (DIAAS), which provides additional information about protein quality [42]. Animal proteins have higher PDCAAS scores than vegetable proteins, without differences in the nutritional value when the patient follows a diversified vegetarian diet [43].

The largest study conducted to assess the effects of plant-based diets, Adventist Health Study 2, enrolled 96,335 participants grouped into five different dietary interventions: non-vegetarian (consuming non-fish meat more than once per month or any meat more than once per week), semi-vegetarian (consuming non-fish meat or any meat more than once per month, but no more than once per week), pesco-vegetarian (consuming fish meat more than once per month but all other meat less than once per month), lacto-ovo-vegetarian (consuming eggs or dairy more than once per month, but fish or all other meats less than once per month), and vegan (consuming eggs or dairy, fish and all other meats less than one time per month) [44]. Vegetarian dietary patterns were associated with a lower mortality compared with the non-vegetarian regimen [44]. Regarding the potential risk of protein malnutrition, the study revealed that the patients who consumed different patterns of vegetarian diets reached or even exceeded the minimum requirements of protein intake [18,44,45].

**Nitrogen balance.** Vegan and vegetarian diets appear to generate a smaller quantity of uremic toxins by changing the proteolytic intestinal bacteria into a Saccharolytic profile with the promotion of the production of short-chain fatty acids to form the intestinal barrier and reduce bacterial translocation and inflammation [45,46].

**Acid-base and electrolytic abnormalities.** Meat consumption is usually associated with increased protein intake; the meat itself, mainly in a highly processed form, accentuates metabolic acidosis in patients with CKD. Acidemia induces protein-energy malnutrition by promoting glucocorticoid secretion and insulin resistance, thus interfering with albumin synthesis [47]. Vegetable proteins are usually pH neutral. Increasing the consumption of vegetables helps to achieve a better control of acid–base balance [47]. In addition, although vegan and vegetarian diets have an increased potassium content, hyperkaliemia is relatively rare, due to the increased fiber content that allows only a small amount of potassium to be reabsorbed [48,49]. Correcting metabolic acidosis improved patient survival and attenuated the progression of CKD [50].

**Bone mineral disorders.** Mineral and bone disorders in CKD are associated with increased cardiovascular and all-cause mortality and a more rapid decline in kidney function [51,52,53]. Plant proteins contain higher levels of phosphorus compared to animal proteins. However, phosphates are stored in a non-absorbable phytate form. Therefore, only one-third of the phosphate from plant foods is bioavailable due to the inability of the human body to metabolize the phytate. LPDs have an additional effect in calcium and phosphate homeostasis by reducing the serum levels of the fibroblast growth factor 23 (FGF 23) by up to 33.5% [54]. About two-thirds of the total phosphates from animal-based foods are bioavailable; consequently, abnormalities of calcium-phosphorus metabolism are more common in patients who consume mostly animal proteins, increasing secondary hyperparathyroidism [6,51,55].

**Arterial hypertension**. Some beneficial effects on blood pressure control, especially on diastolic blood pressure, were also observed [53,56]. Moreover, the benefit appears to be more significant in patients who were compliant with vegan diets. By comparison, these patients had superior control of their blood pressure levels [57,58,59,60,61,62].

The quality of amino acids appears to influence the blood pressure control. The elevated serum levels of methionine and alanine, found in patients who preferentially consumed animal proteins, were associated with a high arterial blood pressure. The increased serum levels of threonine and histidine, frequent among vegan and vegetarian patients, were associated with the optimal control of blood pressure [62].

The benefits of vegetarian LPDs were also observed in diabetic patients. In this regard, it seems that the mean arterial blood pressure could be decreased by 13 mmHg with an optimal multifactorial approach, including a dietary intervention of mostly vegetarian LPD [17].

**Lipid metabolism**. Patients with CKD were frequently associated with abnormalities in lipid metabolism, thus promoting atherosclerosis. Therefore, the control of lipid metabolism represents an important goal. The consumption of soya decreases serum LDL-cholesterol levels and wheat gluten lowers the levels of LDL-cholesterol, triglycerides and uric acid [63,64,65]. Vegetarian diets are beneficial in controlling lipid metabolism, because of a significant reduction in saturated fats, which are replaced with monosaturated fats from herbal or fish oils [64,65,66].

**The role of gut microbiota.** In CKD patients, there is a dysbiotic gut microbiota, characterized by a decrease in commensal bacteria and an increase in uremic toxin-producing bacteria. It seems that gut-derived uremic toxins, such as p-cresol and indoxyl sulfate promote the acceleration of cardiovascular disease [67,68]. Other uremic toxins, such as trimethylamine N-oxide (TMAO) stimulate atherosclerosis and can also increase the risk of mortality in CKD [69]. This imbalance seems to be modified by dietary interventions. In this regard, the fiber content of LPDs, especially in vegan and vegetarian diets seems to reduce protein fermentation and to increase carbohydrate fermentation [70,71]. Compared with omnivores, vegan and vegetarian diets have lower contents of L-carnitine, that may reduce the production of TMAO, and thus reducing the mortality through cardiovascular disease [72].

## 4. Vegetarian LPDs in Special Circumstances

Although LPDs have positive effects on delaying KRT and on obtaining the control of different metabolisms, it should be taken in consideration that there are special patient populations in which LPD seems to be contraindicated [46]. In this respect, we refer to the presence of hypercatabolic conditions, protein-energy wasting, eating disorders or even the short life expectancy of the patients [46].

Socio-economic and cultural barriers could be a hindrance to obtaining adherence to LPDs but can be overcome by individualizing the psychological approach, in addition to nutritional interventions.

Patients with psychiatric disorders represent another challenge in obtaining compliance to nutritional intervention, but with a psychiatric intervention and closer follow-up, adherence to LPD could be achieved due to interdisciplinary communication.

Old age is not a contraindication in adopting LPD, but the close monitoring of elderly patients (considered as over 80 years of age) should be taken into account due to the presence of possible frailty, as well as their frequent association with cognitive disorders. There are few studies that analyze the benefits and risks of LPDs in elderly patients, but the conclusion would be that prescribing a LPD regimen in the elderly also seems to delay KRT without consequences for patient survival [73].

## 5. Conclusions

Low-protein diets are feasible and have beneficial effects in patients with chronic kidney disease, with and without diabetes, both in postponing kidney replacement therapy, but also for a better patient survival on the long term, as these diets are nutritionally safe. The vegetarian-supplemented, very-low-protein diets seem to have an additional positive effect on patient and kidney survival.

Vegetarian and vegan diets seem to be superior to mixed diets and could improve some metabolic disturbances related to chronic kidney disease and optimize blood pressure control.

Protein restriction, including the plant-based, low-protein diets (vegetarian or vegan diets), are nutritionally safe in chronic kidney disease patients, with or without diabetes mellitus, in patients who achieve the required energy intake.
